# Identification of microRNAs as potential cellular monocytic biomarkers in the early phase of myocardial infarction: a pilot study

**DOI:** 10.1038/s41598-017-16263-y

**Published:** 2017-11-21

**Authors:** Mariana S. Parahuleva, Gerhild Euler, Amar Mardini, Behnoush Parviz, Bernhard Schieffer, Rainer Schulz, Muhammad Aslam

**Affiliations:** 1 Internal Medicine/Cardiology and Angiology, University Hospital of Giessen and Marburg, Marburg, Germany; 2 Internal Medicine I/Cardiology and Angiology, University Hospital of Giessen and Marburg, Giessen, Germany; 3 Institute of Physiology, Justus Liebig University, Giessen, Germany

## Abstract

MicroRNA has been increasingly suggested to be involved in vascular inflammation. The aim of this study was to assess the expression profile of miRs as possible novel cellular biomarkers in circulating monocytes in patients with ST-segment elevation myocardial infarction (STEMI). Microarray techniques and TaqMan polymerase chain reaction were used to analyse the global expression of 352 miRNAs in peripheral blood monocytes from healthy donors (n = 20) and patients (n = 24) with acute STEMI. The expression level of miR-143 in monocytes from STEMI patients compared to healthy controls was increased, whereas the expression of miR-1, -92a, -99a, and -223 was reduced significantly. During 3.5 ± 1.5 months of follow-up miR-1 and -223 were back to baseline, whereas miR-92a and -99a return to normal levels over 3 months, but remained lower than healthy controls. Furthermore, monocytic expression of miR-143 was positively correlated with hs-CRP (R^2^ = 0.338; P < 0.031), but not with cTnT. Importantly, treatment of monocytes isolated from healthy individuals with INFγ, but not LPS or TNFα caused an upregulation of miR-143 and downregulation of miR-1. Our findings identify circulating monocytes as putative biomarkers and as novel carriers for the cell-specific transfer of miRs in the early phase of myocardial infarction.

## Introduction

Myocardial infarction (MI) occurs predominantly as a result of acute atherosclerotic plaque rupture with inflammation believed to be a key participant^1,2^. Elevated levels of circulating monocytes provide an expanded pool of inflammatory cells available for recruitment to growing arterial lesions^3^. Blood monocytes instigate inflammation and together with their lineage descendant, mostly only ‘classically polarized’ macrophages, deliver proteolytic enzymes that digest extracellular matrix and potentially render plaque unstable^4–6^. During acute MI, blood monocyte levels rise, and accumulate in the evolving myocardial wound^7,8^. Thus, the organism experiences an acute inflammatory event and systemic response to ischemic injury superimposing the pre-existing chronic vascular inflammatory disease, both of which involve the same inflammatory cell type – the circulating blood monocytes^8^. Furthermore, the systemic inflammatory setting persists several weeks after acute MI and is associated with markedly increased monocyte recruitment and acceleration of pre-existing atherosclerosis^9^.

MicroRNAs (miRs) have emerged as key regulators of several physiological and pathophysiological processes in cardiovascular health and disease^10,11^. They are modulated in diverse cardiac diseases, e.g. in myocardial infarction, heart failure, hypertrophic cardiomyopathy or hypertension, either in the heart tissue or in the circulation^12^. Besides their intracellular function, recent studies demonstrate that miRs can be exported or released by cells and circulate in the blood in a remarkably stable form^13^, and they can be transferred between different cell types^14^. MiRs have been also widely shown to have diagnostic and therapeutic value in vascular inflammatory diseases and vascular cell damage^13^. Acute MI for example induces a distinctive circulating miRs signature and specific circulating miRs have been shown to be associated with the clinical subtype of acute coronary syndrome (ACS) and could be used as biomarker^15,16^. Because miRs play critical roles in the cardiovascular biology and are upstream regulators of gene expression, it would be needful to address their role in systemic response to ischemic injury and vascular inflammation during and after MI, in particular in leukocyte activation and their infiltration into the vascular wall. However, no information is currently available with respect to cellular miRs expression in circulating human monocytes under inflammatory condition during the early phase of acute MI development *in vivo* and this will be the object of this prospective case-control study. To test the hypothesis that MI changes the miR profiles of whole blood subcomponents, including circulating blood monocytes in patients with myocardial ischemia and whether they have any biological action, we serially imaged miR signature of human monocytes during and 3 months after primary MI. We identified abundantly expressed miRs in circulating monocytes from patients with primary acute ST-segment elevation myocardial infarction (STEMI) with microarray techniques and further analyzed selected miRNA candidates by qRT-PCR. Our observations identified cellular monocytic miRNA candidates, which are correlated with early acute phase of MI and vascular inflammation and may help to develop future diagnostic and therapeutic abilities to identify and evaluate acute MI.

## Material and Methods

### Ethics Statement

The protocol for the study and all experimental protocols were approved by the Ethics Committee of the University of Giessen and informed written consent was obtained from all subjects. Furthermore, all methods were carried out in accordance with relevant guidelines and regulations.

### Study population and study design

**Figure 1:**
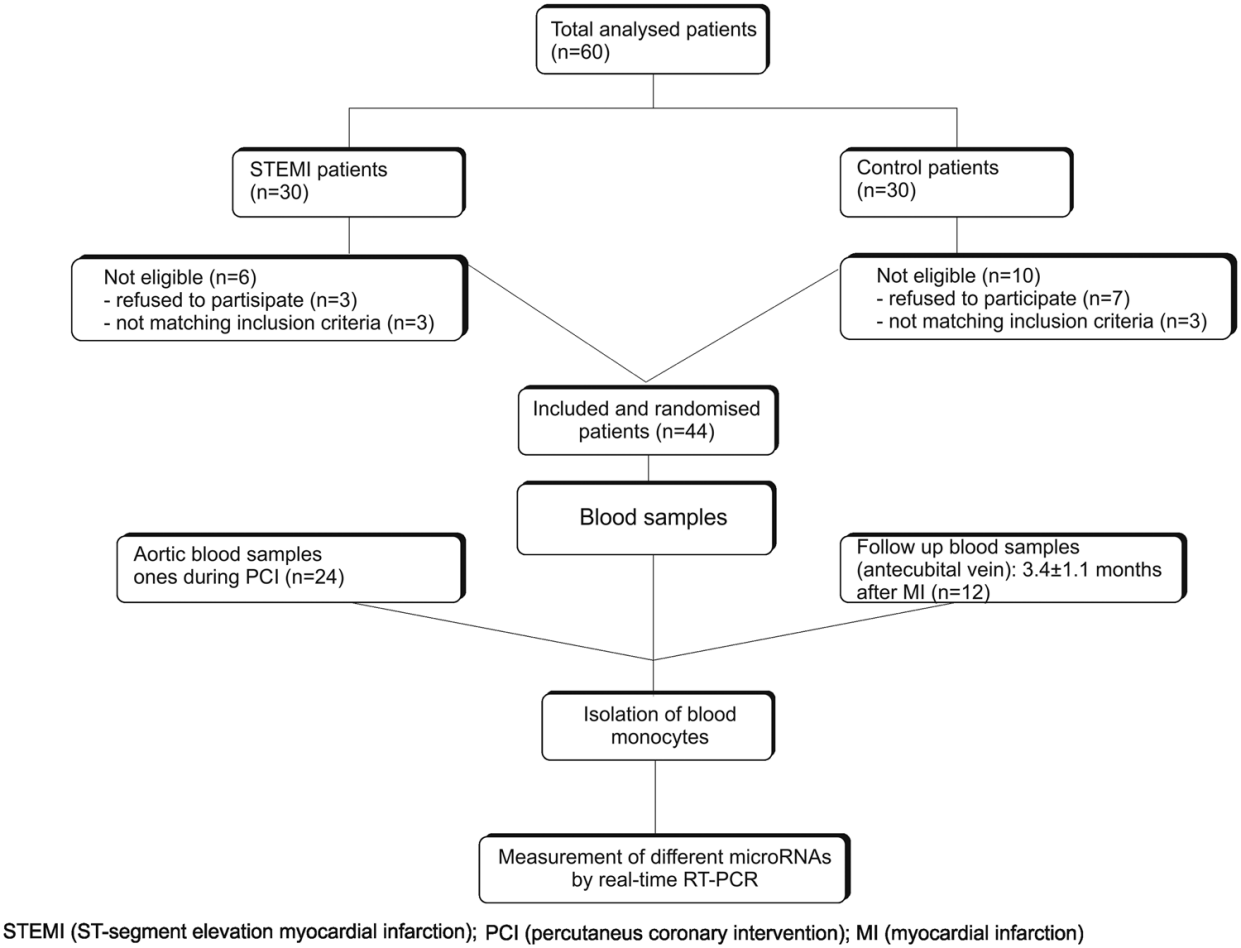
Study design and workflow.

This study is a monocentric, prospective case-control trial and included 24 patients with primary acute ST-segment elevation myocardial infarction (STEMI) admitted to the Department of Internal Medicine I Cardiology/Angiology, University Hospital of Giessen-Marburg. From the initially included 30 patients, 3 patients refused to participate and 3 patients did not match the inclusion criteria (Fig. 1). From the remaining patients (n = 24), included in this study, 12 patients (50%) were lost to follow-up. The mean follow-up period was 3.5 ± 1.5 months after primary STEMI. The mean (±SD) age of the remaining 24 patients was 63.7 ± 10.2 years, with high prevalence of CV risk factors: hypertension (79.5%), hyperlipoproteinaemia (85.4%), diabetes (30.5%), and smoking (19.3%). All patients underwent emergency percutaneous coronary intervention (PCI) and had clinically significant ST-segment elevation with ongoing chest pain for less than 12 hours. Blood samples for simultaneous miRNA and values of cardiac markers determination were collected 7.66 ± 1.84 hours after the onset of symptoms and, again, 3.5 ± 1.5 months later as follow-up (Table 1). The entire list of all 24 cases/ patients can be found as Supplementary Table S1. Treatment of the patients was performed according to the hospital standard routine following the guidelines for acute myocardial infarction with STEMI^17^. We only included male patients in the initial study, in order to eliminate variations caused by hormonal varieties in the female cycle. STEMI was defined as persisting chest pain at rest with ST-segment elevation (≥0.1 mV), T-wave inversion (≥0.1 mV) or (presumed) new left bundle-branch block, and elevation of biochemical markers (creatinekinase (CK), creatinekinase myoglobin binding (CKMB) isoenzyme or cardiac troponin T (cTnT)^18^. Standard procedures for the quantification of CK, CKMB, and cTnT were used (high-sensitivity assays, Siemens Medical Solutions Diagnostics, Germany). Age, gender, BMI (body mass index), blood pressure levels, history of previous myocardial infarction, cardiovascular risk factors including systematic hypertension, diabetes mellitus, smoking, hyperlipoproteinaemia, and family history of CAD, as well as cardiac medications were assessed and recorded at study entry (Table 1). Patients were excluded if (i) they had recent percutaneous coronary intervention (PCI); (ii) they had recent myocardial infarction; (iii) they had previous coronary artery bypass grafting (CABG); (iv) they had coronary artery tree free of significant lesions detected by coronary angiography; (v) they were unwilling to provide written informed consent to participate; (vi) there were contraindications for PCI and/or only indication for CABG. Patients with STEMI undergoing treatment by stent placement were compared to healthy individuals. 20 normal controls were randomly selected from healthy individuals who had contemporaneously visited University Hospital of Giessen for a routine physical examination. All subjects were found to be normal on physical examination, had normal electrocardiograms and routine laboratory blood chemistry without evidence of diseases such as hyperlipoproteinaemia, hypertension, acute cardiovascular or cerebrovascular disease, diabetes mellitus, respiratory tract infections or any clinical evidence of atherosclerosis, and none taking any medication.

**Table 1:** Characteristics of the study groups.

Parameters	control n = 20	STEMI n = 24	P value
Age (years), mean ± SD	57 ± 14.5	63 ± 10.2	ns
Male gender, n (%)	100	100	ns
BMI (kg/m2), mean ± SD	24.1 ± 2.74	28.7 ± 4.74	<0.05
**Risk factors (%)**			
Hypertension	0	79.5	<0.001
Diabetes mellitus	0	30.5	<0.001
CAD in family	0	45	<0.001
Smoking (current smoker)	0	19.3	<0.001
Hyperlipoproteinemia	0	85.4	
Time from symptom onset to blood withdrawal (h)	0	7.66 ± 1.84	<0.001
Number of vessels	—	I. 12.5%	
	—	II. 37.5%	
	—	III. 50%	
**Target vessel / Infarct-related artery (%)**			
LAD (%)	—	62.5	
RCA (%)	—	16.7	
RCX (%)	—	20.8	
**Medication (%)**			
ß-Blockers	0	68.7	<0.001
ACE inhibitors/AT1 Antagonists	0	68.7	<0.001
Diuretics	0	37.5	<0.001
Statins	0	43.7	<0.001
ASS/Clopidogrel	0	43.7	<0.001
**Laboratory values**			
hs-CRP (mg/l)	<5	41.8 ± 15.3	<0.001
Cardiac Troponin T (ng/l)	<0.3	24.5 ± 15.6	<0.001
Creatinkinase (U/l)	<171	453 ± 111	<0.001
Creatinkinase – MB (U/l)	<24	89 ± 19	<0.001
Glucose (mg/dl)	85 ± 1.7	125.5 ± 7	<0.001

Values are mean ± SD. p values indicate the difference between a control group and all other groups. Reference indicates age- and sex-matched normal reference group; smoking, percentage of subject with a history of smoking within the past 6 months; diabetes mellitus, percentage of subjects with ongoing treatment for diabetes mellitus or having a fasting blood glucose ≥126 mg/dl; hypertension, percentage of subjects with a systolic blood pressure ≥140 mm Hg and/or diastolic blood pressure ≥90 mm Hg.

### Blood samples

In order to detect the expression trends of miRs in early phase of AMI, the first blood sample was collected immediately from each acute MI patient after admission to cath lab and insertion of a sheath in the femoral artery. Sample collection time in each case was defined as pain-to-balloon time, which starts with the symptom onset (pain) and ends when a catheter guidewire crosses the culprit lesion and before the procedure of PCI, while the blood samples for simultaneous miRNA and values of cardiac markers determination were collected (Supplementary Table S1). The follow-up blood sample was obtained at 3.5 ± 1.5 months after AMI and stent implantation. The blood samples at 3.5 ± 1.5 months after AMI and from healthy individuals were collected by venepuncture. In all cases, 20 ml peripheral blood samples were obtained by direct venipuncture and immediately transferred into ethylenediaminetetraacetic acid (EDTA).

### Peripheral Blood Monocyte Preparation

Peripheral blood monocytes were isolated from peripheral blood using the RosetteSep®-Kit according to manufacturer’s instructions as described previously^19^. Briefly, 250 ml anticoagulated blood was centrifuged for 10 minutes at 2000 rpm. Plasma was taken off and frozen at −80 °C for potential further experiments. Haematocrit with RosetteSep Cocktail and 2% BSA in PBS/EDTA were layered over FICOLL and centrifuged for 20 minutes at 2200 rpm without brake. When centrifuged this way unwanted cells pellet with the red blood cells and monocytes form a layer between this pellet and plasma. The purity (>92%) of monocytes was determined by FACS analysis. Freshly isolated CD14^+^ cells were snap-frozen and stored at −80 °C until RNA isolation.

### RNA-Isolation

Afterwards, total RNA was extracted from monocytes using the Roti®-Quick-Kit (ROTH, Karlsruhe, Germany) following the manufacturer’s instructions and as described previously^18^. RNA concentrations were measured using an Eppendorf® BioPhotometer. In addition, we used the SABiosciences RT² qPCR-Grade miRNA Isolation Kit (SABiosciences Corporation, Frederick, MD, USA) to enrich miRNA from 40 µg of total RNA of each sample according to manufacturer’s instructions. This kit combines a phenol/chloroform-based extraction method with a silica membrane spin column technology. MiRNA was then reverse transcribed using the SABiosciences RT² miRNA FirstStrand Kit:331401 according to the manufacturer’s protocol and as described previously^20^.

### MiRNA expression profile

Samples were analyzed with the SABiosciences Human miFinder RT² microRNA PCR Array, 96-well (SABiosciences Corporation, Frederick, MD, USA). One sample per group was analyzed using four different miRNA PCR Array-plates, each containing different miRNAs and snord 44 as reference gene. Since most interesting results could be seen on Human miFinder/genome plate 1 (SABiosciences Corporation, Frederick, MD, USA), three more samples of each group were analyzed with this plate.

### Real-time reverse transcriptase polymerase chain reaction (real-time RT-PCR)

Relative miRNA quantification of specific miRNAs in tissue and blood samples was performed by real-time RT-PCR using RT² SYBR® Green qPCR Master Mix (SABiosciences Corporation, Frederick, MD, USA) and CFX 96 real-time system Bio-Rad (Bio Rad, Munich, Germany) according to the manufacturer’s protocol. Briefly, a total of 20 µl were added to each well, containing 1 µl of reverse transcribed miRNA, 1 µl of miRNA qPCR Assay primer, 8 µl of ddH2O and 10 µl of master mix per well. Thermal protocol contained 10 min of denaturation at 95 °C followed by 40 cycles of 95 °C for 15 sec, 55 °C for 40 sec and 72 °C for 30 sec for hybridization and elongation. Real-time RT-PCR reactions were performed in triplicates. MiRNAs were considered as present when CT-values (threshold cycle) were lower than 30. Snord 44 was used as housekeeping gene for normalisation. Gene expression was assessed using the 2^−ΔΔCt^ calculation method as described previously^19,20^.

### Monocyte cell culture and treatment *in vitro*

Human peripheral blood mononuclear cells were isolated from buffy coats obtained from University hospital blood transfusion centre donated by healthy volunteers and cultured in serum free monocyte cell culture medium (Invitrogen, Darmstadt Germany) as described previously^19^. Cells were allowed to attach for 2 h followed by treatment with inflammatory agents (LPS 10 μg/ml, TNFα 10 ng/ml, or IFNγ 50 ng/ml) in the monocyte medium for 12 h. Afterwards, total RNA was isolated using miRNeasy kit (Qiagen GmbH, Hilden Germany) according to manufacturer’s instructions and miR analysis was performed by qPCR as described before.

### Data presentation and statistical analysis

Data are presented as box plot with median (25th/75th percentiles) and whiskers (Tukey). Data were found to be not normally distributed according to D’Agostino & Pearson omnibus normality test and were compared using Kruskal-Wallis test followed by Dunn’s corrections for multiple comparisons. The alpha value was 0.05 and the adjusted p-values are presented in each graph. Linear regression analysis was performed to determine the relationships between cardiac Troponin (cTnT) as well as hs-CRP and the different miRs using the logarithmic masses. All statistical calculations were performed using the statistical package GraphPad Prism, version 6.05 (GraphPad Software, Inc., USA). All hypotheses were 2-tailed, and P ≤ 0.05 was considered statistically significant.

## Results

### Selection strategy of relevant miRs related to patients in the early phase of myocardial infarction

To determine the influence of myocardial infarction (MI) on the levels of miRNA expression in circulating blood monocytes, we performed initially a miRNA profile using miRNA PCR array covering 352 human miRNAs with in each case RNA isolated from n = 4 patients with MI and n = 4 healthy controls. A detailed description of the patient’s and healthy individual’s characteristics is given in Table 1. Selection of miRNAs was first conducted by careful in silico analysis, studying the literature for relevant miRs associated with MI^16,21,22^. We also screened for miRNAs related to cardiovascular pathogenesis including endothelial injury, endothelial activation, and vascular inflammation^21^. We then performed a database screening (TargetScan.org, miRBa-se.org, microRNA.org) to determine relevant miRNA biomarker candidates according to their predicted interactions with molecules of interest. Following this analysis, we identified 16 miRNAs of interest for MI (Table 2). Quantification revealed that multiple miRNAs were significantly downregulated in patients compared with healthy controls (p < 0.05; Table 2). Although the absolute number of significantly downregulated miRs prevailed, some monocytic miRNAs were identified demonstrating increased levels in patients (Table 2). Interestingly, most if not all of the highly expressed and significantly downregulated miRNAs in patients with MI are known to be expressed in the vascular wall, particularly in endothelial cells. These endothelial miRNAs include miR-92a, miR-21, miR-29, miR-19b, and members of the let-7 family (let-7f) (Table 2). In contrast to the low levels and profound downregulation of endothelial and vascular miRNAs, cardiac- and smooth muscle-expressed miRNAs were detected particularly at high levels. Interestingly, the established smooth muscle– enriched miRs miR-143 was significantly upregulated in patients compared with healthy volunteers (Table 2). miRs related with inflammation (miR-155, miR-181b) were not profoundly regulated in patients with primary MI (Table 2). Finally, from a miRNA profiling in a matched derivation case-control cohort, 11 miRs were carried over to the validation phase and were chosen for further investigation using real-time RT-PCR.

**Table 2:** Up- and Down-regulated miRs in patients in the early phase of myocardial infarction versus healthy controls.

miRNA	Fold change	P-values
let-7f	−7.5	0.001
miR-1	−4.7	0.01
miR-9	−2.4	0.05
miR-19b	5.7	0.01
miR-21	−9.9	0.001
miR-22	5.2	0.001
miR-29b	−6.8	0.001
miR-92a	−2.5	0.05
miR-96	−4.2	0.001
miR-99a	−3.3	0.01
miR-143	12.7	0.001
miR-181b	−2.7	0.05
miR-223	−9.8	0.001
miR-133b	−2.2	0.05
miR-29a	−4.2	0.01
miR-155	−2.3	0.05

miRs were detected with the SABiosciences Human miFinder RT² microRNA PCR Array in healthy controls (n = 4) or patients with STEMI (n = 4). Relative mRNA quantification was performed and the fold change in the target miR, normalized to the internal control (Snord 44) and relative to the expression in healthy controls, was calculated and presented (cut off > 2). Significance was assumed at p < 0.05 (corrected p-values).

### Differentially regulated miRNAs in monocytes in myocardial infarction patients

**Figure 2:**
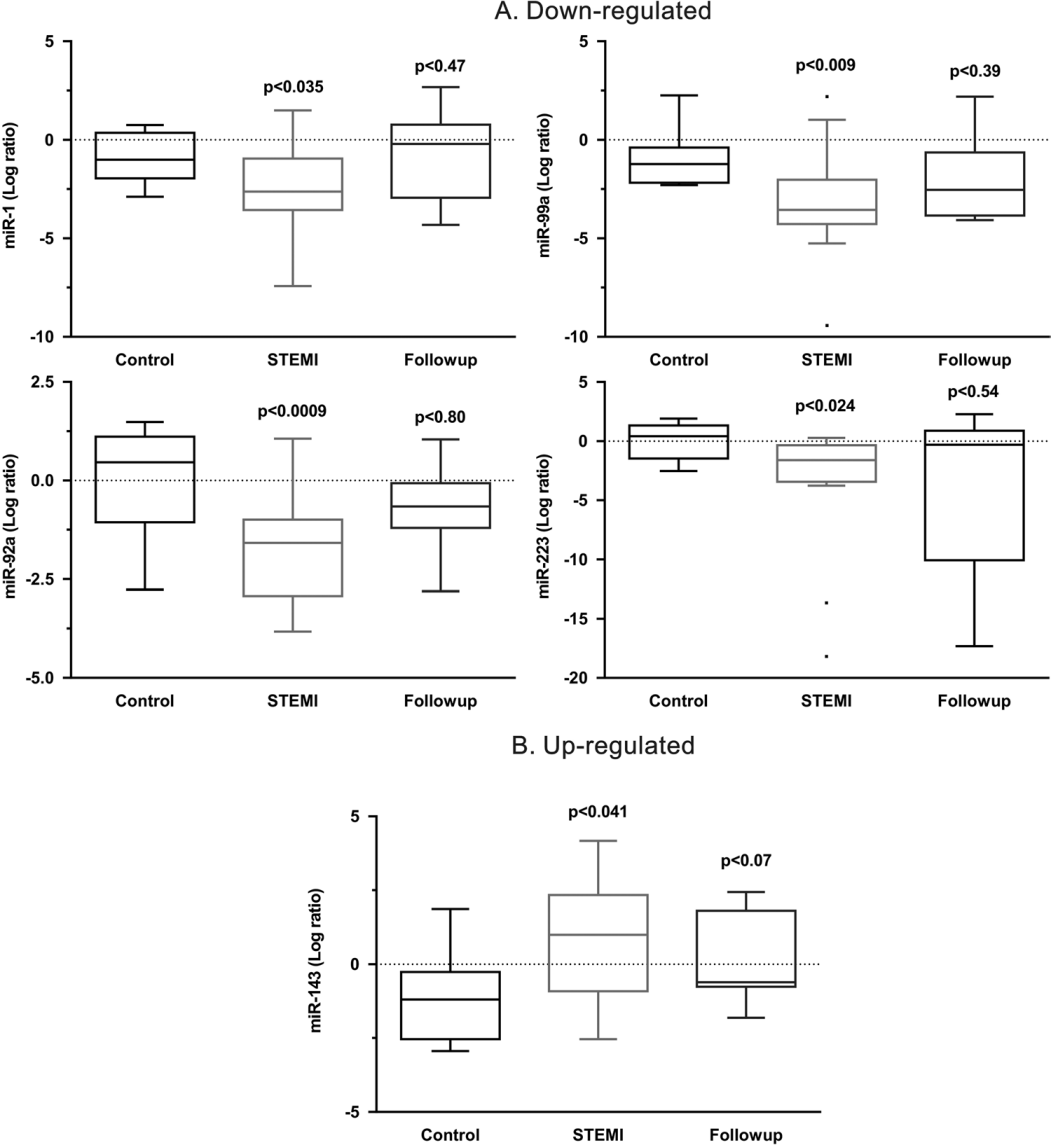
RT-qPCR-analysis of expression of miRs in circulating monocytes from patients with STEMI (n = 24), follow up (n = 12), and healthy controls (n = 20): (**A**) miRs down-regulated in STEMI patients vs. control, (**B**) miRs up-regulated in STEMI patients vs. control. Snord44 was used as reference gene for normalization and the relative miRNA expression was calculated using the 2^−ΔΔCT^ method. Data are presented as box plot with median (25th/75th percentiles) log ratios (Tukey) and compared using Kruskal-Wallis test followed by Dunn‘s corrections for multiple comparisons. The alpha value was 0.05 and the adjusted p-values compared to control group are presented in each graph.

**Figure 3:**
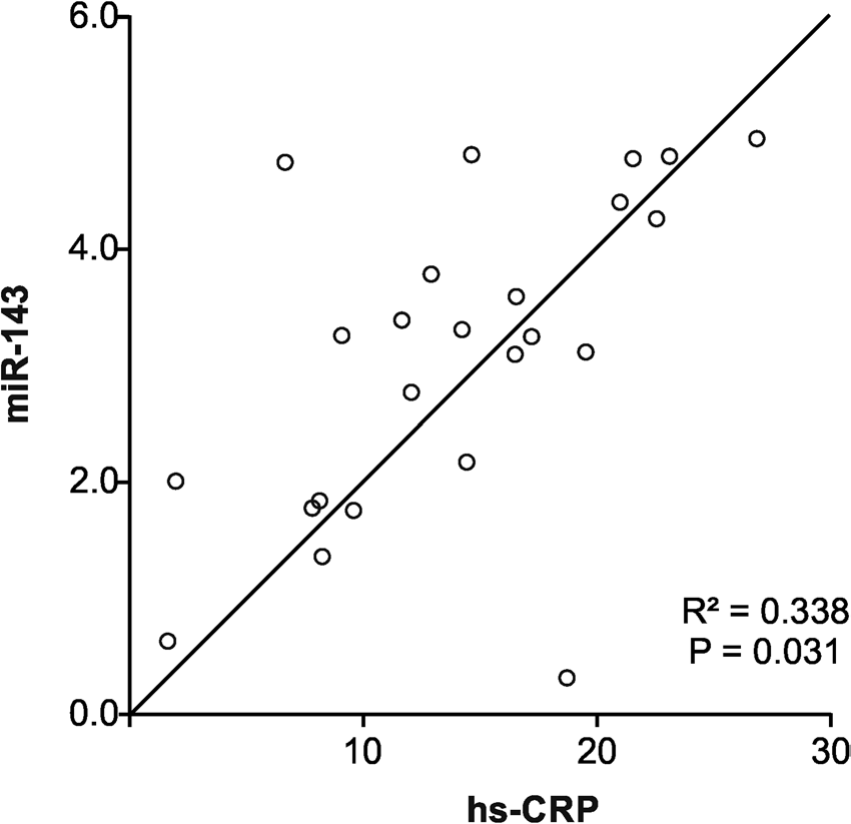
Linear regression analysis of correlation between monocytic miR-143 and baseline levels of hs-CRP.

Based on the microarray data, 11 miRNAs; let7f, miR-1, miR-19b, miR-21, miR-29a, miR-29b, miR-92a, miR-99a, miR-133b, miR-143, and miR-223, which are linked to cardiovascular function/regeneration were selected for further analysis in the STEMI patients (n = 24), at the time of onset and 3.5 ± 1.5 months of follow-up after primary acute MI (n = 12) and compared with healthy controls (n = 20). Expression profiles of the miRNAs were verified using TaqMan real-time qPCR. As shown in Fig. 2, out of 11 miRs, 5 miRs were reliably detected and significantly verified in the majority of samples: miR-1, miR-92a, miR-99a, miR-143, and miR-223. According to their expression pattern across the patient’s samples the investigated miRs could be classified into 3 different groups, (A) unchanged expression, (B) down-regulated expression vs. control and (C) up-regulate expression vs. control (Fig. 2). The expression of miR-1, miR-92a, miR-99a, and miR-223 was down-regulated while that of miR-143 was up-regulated. Interestingly, the expression of these miRNAs returned back towards normal levels in patients with 3.5 ± 1.5 months of follow-up. A follow-up investigation was performed to determine whether the monocytic miRs levels in STEMI patients are associated with prognosis after receiving appropriate treatments. Among the 24 STEMI patients, all patients received emergency percutaneous coronary intervention (PCI), and blood samples were collected immediately after admission to University Hospital of Giessen-Marburg and before the procedure of PCI, and the other follow-up blood samples were obtained at 3.5 ± 1.5 months after STEMI and stent implantation. As shown in Fig. 2, the expression levels of monocytic miR-143 declined and expression levels of miR-1, miR-92a, miR-99a, and miR-223 gradually increased towards their baseline values after 3.5 ± 1.5 months of follow-up. These observations indicated that expression levels of miR-1, miR-92a, miR-99a, miR-143, and miR-223 may gradually return to normal levels (over 3 months) after receiving appropriate treatment. Furthermore, no primary STEMI patients experienced an adjudicated major cardiovascular event, defined as cardiac death or recurrent myocardial infarction within 3.5 ± 1.5 months of follow-up. In order to confirm whether the levels of these monocytic miRs are associated with plasma cTnT and hs-CRP in STEMI patients, correlation analysis was performed. The results showed that miR-143 (R^2^ = 0.338; P < 0.031) exhibited significant positive correlation with hs-CRP (Fig. 3), whereas no correlation was found between examined miRs and cTnT (data not shown). However, there was a trend towards positive correlation of miR-223 with hs-CRP (R^2^ = 0.156; P < 0.058), which remain not significant. With regard to the interaction between the levels of miRs in blood monocytes and cardiac risk in patients with STEMI, we have investigated the combined endpoint of mortality and non-fatal myocardial infarction for the initial 24 h and 72 h periods (n = 24), including periinterventional events and for the 3.5 ± 1.5 months follow-up (n = 12). However, in this pilot study any adverse cardiovascular events in the patients with STEMI has been detected and severity of the disease between the groups with up- and downregulated cellular monocytic miRs did not differ.

### *In vitro* analysis of miRNA expression in isolated human monocytes

**Figure 4:**
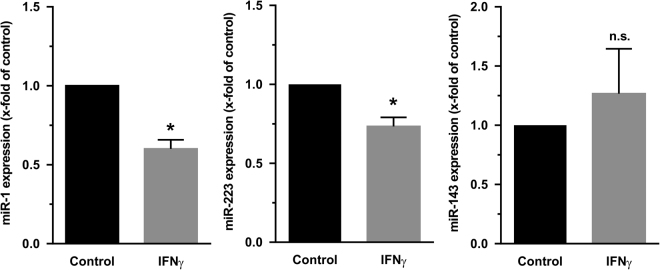
RT-qPCR-analysis of expression of miRs in isolated monocytes treated with IFNγ: Freshly isolated human blood monocytes were treated with IFNγ (50 ng/ml) for 12 h and analyzed for the expression of different miRs as described in results. Snord44 was used as reference gene for normalization and the relative miRNA expression was calculated using the 2^−ΔΔCT^ method. Data are presented as fold change ± SD compared to vehicle treated controls which were taken as 1 (n = 3; p < 0.05 vs control) n.s.: not significantly different from control.

In order to investigate whether simulated inflammatory conditions may also induce the changes in miRNA expression in isolated human monocytes *in vitro*, the monocytes were treated with tumor necrosis factor alpha (TNFα), lipopolysaccharide (LPS) or interferon gamma (IFNγ). The expression of the 5 miRs differentially regulated in monocytes from STEMI patients was analyzed in these samples. Interestingly, TNFα or LPS were unable to induce changes in the expression of the miRs analyzed; however, IFNγ was able to down-regulate the expression of two miRs, miR-1 and miR-223, significantly (Fig. 4). Likewise, there was a trend towards up-regulation of miR-143 in monocytes treated with IFNγ, though it did not reach the level of significance.

## Discussion

Although the mortality rate of myocardial infarction (MI) has much improved since rapid revascularization of occluded coronary arteries became common practice, MI remains to be one of the leading causes of death and chronic heart failure^17,18^. Tissue damage from MI occurs as a result of the initial ischemic event, determined primarily by its duration, and then subsequent injury resulting from reperfusion^18^. Since miRNAs have been implicated as crucial regulators of gene expression associated with MI and remodeling^23^, miRNA expression might alter during the various biological processes in MI in both the border zone and remote zone^24^. MiRNA expression is altered in the infarcted myocardium and expression of miRs changes temporally in response to MI within two months after MI, suggesting that altered expression of miRNAs is associated with progression of MI^25^. In this regard, it could be suggested that the temporally altered expression of miRs correlated with multiple stages post-MI, such as early stage apoptosis or later stage growth promotion^26^. Interestingly, our data indicated that expression of miR-143 in circulating blood monocytes is upregulated in the early phase of MI, but does not increase consistently after PCI treatment post-MI and returns to base line after 3 months of follow-up (Fig. 2). Additionally, we identified that four other miRs (miR-1, miR-92a, miR-99a, and miR-223) differentially downregulated in circulating monocytes in acute MI patients and returned to basal levels after 3 months (Fig. 2). This indicates that the early phase of MI induces transient aberrant regulation of monocytic miRs expression, which is possibly unique, and may be considered as a new clinical target.

Most of the studies have mainly focussed on the analysis of free circulating miRNAs in the serum being relatively easy to carry out^11–15^. However, additional information is required to further understand the miRNA species in whole blood subcomponents, such as plasma, platelets and leukocytes. In this regard one important component, the monocytes that are one of the major factors in initiation and progression of atherosclerosis and development and acceleration of MI are mostly omitted or less studied. Monocytes and their derivative cells express certain set of miRs, which are modified in the presence of certain disease conditions. Some recent studies have reported the expression profile of miRNAs in human monocytes mainly from healthy individuals^27,28^. But little data are available on the expression profile of miRs in circulating blood monocytes from acute MI patients. Thus, our study is the first to describe early dysregulation of miRs in monocytes within the first 24 h after onset of acute MI and their modulation during the early inflammatory response to MI in man.

The present study is the first to describe downregulation of miR-1 in circulating monocytes after acute MI, a miRNA that is normally highly expressed in skeletal muscle as well as in the heart. The monocytic miR-1 expression returned completely to the base level within 3 months after acute MI. Interestingly, elevated plasma levels of miR-1 have been reported in patients suffering from MI, however, no correlation between its plasma levels and severity of MI could be determined^29–32^. Likewise, increased cardiac expression of miR-1 has been reported after MI^33^. But again no correlation between miR-1 expression levels and adverse outcome could be established^34^. However, a positive correlation was found between miR-1 levels and infarct volume, as well as trend for correlation with left ventricular ejection fraction^34^. In our study, we now identified downregulation of miR-1 in circulating monocytes, although in plasma it was found upregulated after MI^33^. Explanations for this discrepancy may lie in a different cellular source of miR-1. Also in cardiac arrhythmias contrary expression levels of miR-1 have been found, either reduced in atrial tissue or increased in ventricular tissue^35^. During MI, miR-1 can be released from injured cardiomyocytes, and thereby enter the circulation. Then increased miR-1 levels in plasma can be expected. But in our study, miR-1 is decreased in monocytes. Interestingly, Wronska *et al*. already discussed that during AMI, release of miRs from injured cardiac tissue elevates miR plasma levels, whereas the level of vascular miRs are decreased in coronary artery diseases^12^. Since miR-1 targets connexin43 and Kir2.1, a component of the cardiac K + channel, its cardiac function is related to arrhythmias^12^. However, if and how monocytic miR-1 expression is involved in cardiac function in MI or if it is just a potential diagnostic marker has to be analyzed in further studies.

Angiogenesis is a critical component in post-MI early tissue repair that participates in limiting the infarct size and reducing myocardial apoptosis^36^. MiR-92a is the most widely studied miRNA for inhibition of angiogenesis after MI. Like miR-1, the plasma levels of miR-92a are elevated in STEMI patients^34^. PCI therapy may suppress such up-regulation, and survival rate is higher in patients showing down-regulation of miR-92a^37^. In a mouse model, antagomir-92a improved left ventricular function, reduced infarct size, attenuated apoptosis, and increased angiogenesis in the border zone^36^. Shortly after MI, various circulating monocytes subsets (CD14^+^CD16^−^in humans) are inflammatory and accumulate in the heart with peak around day 3. These cells are centrally involved in inflammatory tissue remodeling, resolution of inflammation during post-MI healing, and left ventricular remodeling^38^. Therefore, downregulation and inhibiting of miR-92a in circulating inflammatory monocytes subsets after MI may potentially be important in improving remodeling and neovascularization after MI. However, the monocyte expression of miR-92a in patients with MI has not been reported, yet. Our data demonstrate downregulation of miR-92a expression in monocytes after acute MI that returns to the base line after 3 months of follow-up (Fig. 2). Thus, downregulation of miR-92a expression in circulating monocyte in the early phase of MI may serve as a natural protection mechanism. Once recruited in the infarct border zone from the bloodstream, these monocytes could significantly reduce infarct size and improve the recovery of cardiac function in the acute ischemic disease setting.

To test whether cytokines involved in the response to acute heart ischemia are also effective at downregulation of miR-92a expression, human monocytes were stimulated with TNF-α or IFNγ. Both proinflammatory cytokines were ineffective (data not shown), which consider that downregulation of monocytic miR-92a during the early phase of MI seems not be a result of inflammatory conditions. Conversely, IFNγ, but not TNF-α, downregulated the miR-1 expression in human monocytes. Thus, the circulating blood monocytes are not the main source of miR-1 during the early phase of MI under inflammatory setting. Our data suggest that downregulation of monocytic miR-92a, but not of miR-1 might have potential to repair the infarcted myocardial tissue. Thus, approaches that downregulate the miR-92a in human circulating monocytes after acute MI are promising therapeutic applications in the prevention and treatment of STEMI patients as well as in the improvement of the prognosis of acute MI. MiR-223 has been shown to regulate differentiation of monocytes to macrophages, since treatment of monocytes with miR-223 antagomirs prevented PMA or GM-CSF induction of macrophage markers and reduced adherence of monocytes^39^. We now demonstrate downregulation of miR-223 in monocytes from patients with acute MI, thereby indicating that these monocytes counteract the infiltration of macrophages into the damaged tissue. As inflammatory processes are considered to be protective in the early process of MI, the downregulation of miR-223 may be detrimental to heart repair in this situation. Downregulation of miR-223 has also been shown in circulation of patients with a high risk to develop MI^40^. In this study origin of miR-223 was mainly attributed to circulating platelets. But we now define monocytes as another source of reduced miR-223 expression in MI. Similar to our study, Yang *et al*., identified a remarkable reduction of miR-99a in AMI patients^41^. Whereas we detected downregulation in monocytes Yang *et al*. described downregulation in plasma samples, which was closely associated with the severity of the MI. In neonatal cardiomyocytes miR-99a downregulation can be provoked by hypoxic conditions^42^. And intra-myocardial injection of miR-99a attenuated heart remodeling and improved cardiac performance after myocardial infarction. Furthermore, miR-99a modulates the inflammatory response of endothelial cells and may thereby modulate atherosclerotic processes that are predictors of MI^43^. These findings all together indicate a prevailingly protective role of miR-99a in ischemic heart disease. Prevention of miR-99a downregulation may therefore become an interesting therapeutic target.

The only miR that has been found upregulated in monocytes in our study is miR-143. Induction of miR-143 has been found in peripheral blood mononuclear cells of sepsis patients, indicating its induction under inflammatory conditions, as they may also occur in MI^44^. Also we could detect miR-143 upregulation in monocytes from healthy individuals upon treatment with pro-inflammatory IFNγ. Function of this miR has been attributed to atherosclerosis. The Dimmeler group showed induction of miR-143 by shear stress in endothelial cells^45^. This miR can be sequestered in exosomes and reduces plaque size in animal models of atherosclerosis. Also monocytes are able to release miR-143 into the circulation by secretion of exosomes and may thus modulate coronary artery disease in a similar way as when synthesized in endothelial cells^46^.

Interestingly, three of the identified miRs are related to inflammation. We could demonstrate down regulation of the two anti-inflammatory miRs (miR-99a and miR-223), as well as up regulation of the pro-inflammatory miR-143 in monocytes of AMI patients. This indicates a strong pro-inflammatory situation in these patients. All three miRs go back to baseline after PCI, indicating relief of inflammation and thus a good prognosis for reduced disease progression.

Unfortunately, we were unable to demonstrate a definite mechanism of interaction between ischemic heart disease and miRs. As described by Das and Halushka, miRs can shuttle between different vascular and cardiac cell types, thereby increasing the complexity of cell-to-cell regulatory mechanisms^14^. Mainly miR transfer between endothelial cells, fibroblasts, VSMCs and cardiomyocytes have been shown, or from monocytes to endothelial cells. However, since during acute MI, blood monocyte levels rise, and accumulate in the evolving myocardial wound^7,8^. monocytes move into the proximity of cardiomyocytes, thereby increasing the probability of miR transfer between these cell types. Whether differential abundance of monocytic miRs is a result of different release- or uptake- mechanisms or modulated intracellular production has to be determined in future studies. In fact, miRs are known to be encoded from their own promoters inside exonic regions or introns of coding transcripts^10,11^. In addition, it has been reported that expression of miRs could be regulated on multiple levels, including the transcriptional and post-transcriptional level^13^. Thus, the interaction between paracrine factors and miRs is complex and remain unclear. We believe that understanding of this cross-talk is critical and therefore, we are planning further studies to investigate this.

### Limitations

We recognize that our small sample size limits generalizability given the complexity of miRNAs within the biological system in addition to the diverse genetic, social, and treatment characteristics of patients presenting with an acute coronary syndrome (ACS). We are also aware that the differences in patient demographics may also affect these study results. Furthermore, these miRs may reflect pathophysiological mechanisms relevant for clinical outcome and may be further examined for improvement of risk stratification in patients with STEMI. Thus, future studies including larger and more diverse patient populations will be needed to validate our hypothesis-generating findings. We are also aware that it would be ideal to have better information about the timing of symptom onset relative to miRNA quantification. The specific time of this up- or downregulation still needs to be further evaluated. Unfortunately, little information is available with respect to pre-hospital delay in presentation or duration of symptoms, limiting our ability to precisely identify miRNA dysregulation with respect to symptom onset. Future studies will include evaluating monocytic miRNA profiles in well defined patient populations and at different time points during the peri-infarct period.

## Electronic supplementary material

Supplementary Table S1
