# Publisher Correction: Identification of microRNAs as potential cellular monocytic biomarkers in the early phase of myocardial infarction: a pilot study

**DOI:** 10.1038/s41598-018-23598-7

**Published:** 2018-04-11

**Authors:** Mariana S. Parahuleva, Gerhild Euler, Amar Mardini, Behnoush Parviz, Bernhard Schieffer, Rainer Schulz, Muhammad Aslam

**Affiliations:** 1 Internal Medicine/Cardiology and Angiology, University Hospital of Giessen and Marburg, Marburg, Germany; 2 Internal Medicine I/Cardiology and Angiology, University Hospital of Giessen and Marburg, Giessen, Germany; 3 Institute of Physiology, Justus Liebig University, Giessen, Germany

Correction to: *Scientific Reports* 10.1038/s41598-017-16263-y, published online 21 November 2017

In the original version of this Article, the author Mariana S. Parahuleva was incorrectly indexed. This error has now been corrected.

