# Supplementary material for: Identification of microRNAs as potential cellular monocytic biomarkers in the early phase of myocardial infarction: a pilot study
Source: Sci Rep. 2017 Nov 21;7:15974. doi: 10.1038/s41598-017-16263-y (PMC5698316; doi:10.1038/s41598-017-16263-y)
Supplement: Supplementary file 1 — Supplementary Table S1 [file 41598_2017_16263_MOESM1_ESM.pdf]

Identification of microRNAs as potential cellular monocytic biomarkers in the early phase of myocardial infarction: a pilot study

Mariana Parahuleva[1, 2, *], Gerhild Euler[3], Amar Mardini[1], Behnoush Parviz[2], Bernhard Schieffer1, Rainer Schulz[3], Muhammad Aslam[2]

Internal Medicine/Cardiology and Angiology, University Hospital of Giessen and Marburg, Location Marburg[1]; Internal Medicine I/Cardiology and Angiology, University Hospital of Giessen and Marburg, Location Giessen[2], Germany; Institute of Physiology, Justus Liebig University, Giessen, Germany[3]

Patient	Pain-to-balloon sample collection time
1	8
2	5
3	7
4	10
5	8
6	7
7	8
8	9
9	6
10	5
11	7
12	8
13	8
14	7
15	10
16	9
17	7
18	7
19	6
20	6
21	8
22	14
23	7
24	7

Suppl. Table. Pain-to-balloon sample collection time

Pain-to-balloon sample collection time in each case was defined as interval starts with the symptom onset (pain) and ends when a catheter guidewire crosses the culprit lesion in the cath lab, while the blood samples for simultaneous miRNA and values of cardiac markers determination were collected.
